# Brief report: Validity and reliability of the Nigerian Autism Screening Questionnaire

**DOI:** 10.1177/13623613221080250

**Published:** 2022-03-09

**Authors:** Muideen O Bakare, Thomas W Frazier, Arun Karpur, Amina Abubakar, Moses Kachama Nyongesa, Paul Murimu Mwangi, Pamela Dixon, Izma Khaliq, Natalie K Gase, Jonathan Sandstrom, Nwanze Okidegbe, Michael Rosanoff, Kerim M Munir, Andy Shih

**Affiliations:** 1 Federal Neuro Psychiatric Hospital, Nigeria; 2 Childhood Neuropsychiatric Disorders Initiatives (CNDI), Nigeria; 3 Ike Foundation for Autism (IFA), Nigeria; 4 World Psychiatric Association (WPA), Switzerland; 5 John Carroll University, USA; 6 Autism Speaks, USA; 7 Aga Khan University, Kenya; 8 KEMRI-Wellcome Trust Research Programme, Kenya; 9 Columbia University, USA; 10 Harvard Medical School, USA

**Keywords:** autism spectrum disorder, cultural adaptation, epidemiology, Nigeria, screening

## Abstract

Informant-report measures for screening symptoms of autism spectrum disorder (ASD) and other neurodevelopmental disorders (NDDs) are needed for low-resource settings if early identification is to be prioritized because early developmental concerns are likely to be expressed by parents and other caregivers. This paper describes the initial psychometric evaluation of the Nigeria Autism Screening Questionnaire (NASQ). Parents and other caregivers completed the NASQ on 12,311 children ages 1 to 18 in a Nigerian population sample as part of the World Bank National General Household Survey conducted in the country in 2016. Factor analyses indicated a parsimonious three-factor structure with social communication/interaction, repetitive sensory motor, and insistence on sameness dimensions. Measurement invariance was excellent across age and sex. Reliability of the subscales and total scale was good, and item response theory analyses indicated good measurement precision in the range from below average to high scores, crucial for screening, and tracking ASD symptoms. Studies with gold standard ASD diagnostic instruments and clinical confirmation are needed to evaluate screening and diagnostic accuracy. The NASQ appears to be a reliable instrument with a clear factor structure and potential for use in screening and tracking ASD symptoms in future Nigerian samples.

Autism spectrum disorder (ASD) is a neurodevelopmental condition with considerable etiologic and phenotypic heterogeneity (Lord et al., 2020; Muhle et al., 2018). Recent estimates of ASD prevalence across the globe suggest that approximately 0.5%−2% of children meet diagnostic criteria (Alshaban et al., 2019; Diallo et al., 2018; Elsabbagh et al., 2012; Maenner et al., 2020; Zhou et al., 2020). Existing data indicate that early intervention, contingent on early detection, improves cognitive, functional, and symptom outcomes (Dawson et al., 2010; Hardan et al., 2015; Rogers et al., 2019). Even in the western world where awareness of autism has been greatly increased over the last 15 years, the average age of diagnosis remains above 3 years of age, when the vast majority of cases can be reliably identified by 24 months (Lord et al., 2006, 2022). Unfortunately, delayed detection, often due to inadequate screening (Siu et al., 2016), and difficulty in accessing early intervention programs, can delay intervention start by as much as 3 years (Oswald et al., 2017). In low-resource countries, the situation is even more dire, where few countries have sufficient early identification and intervention infrastructure to maximize developmental outcomes. There is a great need for measures that are appropriate for specific low-resource populations and that can be implemented at scale (Patel et al., 2018).

Screening for risk of ASD can effectively increase early identification of ASD in resource-limited settings. In the United States, early screening has helped to lower the age of diagnosis significantly over the last 20 years, and disparities in early diagnosis among racial/ethnic groups are closing (Maenner et al., 2020). In sub-Saharan Africa, there are many barriers to establishing formal screening practices in primary care systems, including low knowledge about autism in many healthcare workers (Bakare et al., 2009; Igwe et al., 2011), the presence of many languages/dialects and a large rural population making access to primary and specialty care very challenging (Franz et al., 2017; Oshodi et al., 2016; Ruparelia et al., 2016), and the lack of psychometrically sound autism symptom instruments developed specifically for broad implementation in the Nigerian context (Bakare et al., 2009; Oshodi et al., 2016).

Thus, there is a great need for brief, reliable, culturally sensitive, and feasible approaches for mapping the risk of ASD in the population to develop strategies for outreach for diagnostic services to children expressing behaviors akin to ASD and other developmental disorders. To fill this need, the Nigerian Autism Screening Questionnaire (NASQ) was created and added to a national World Bank− supported General Household Survey (GHS). Rather than choosing an existing freely available screening tool, the NASQ was developed based on a desire to cover *DSM*-5 criteria (American Psychiatric Association, 2013), to ensure coverage of the full range of presentations for broad application including the potential tracking symptom change, and to ensure that the measure was culturally sensitive and included items that would be relatable to the Nigerian context. Given that establishing diagnostic validity and screening utility is time- and labor-intensive and expensive and would only be justified if initial psychometric properties are favorable, the primary aim of this preliminary investigation was to begin the process of psychometric evaluation of the NASQ. Specifically, the study identified optimal factor structure, evaluated measurement invariance (equivalence) across age and sex, and estimated classical test theory internal consistency reliability and item response theory conditional reliability. Measurement invariance across age is particularly important because initial use of the NASQ is anticipated from ages 1−18 due to the ongoing need to identify older children and adolescents who have not been previously screened or diagnosed in this population.

## Method

### Participants

The NASQ was implemented in the household questionnaire in the post-harvest phase of the Wave 3 GHS-panel data collection. The sample of 5000 households was determined to be nationally representative for the World Bank’s survey aimed to determine social and economic conditions of people in Nigeria. Sampling strategies to ensure generalizability of data to various geographical zones, representing the proportional distribution of urban and rural households was implemented. Additional information on sampling methods for the GHS Wave 3 data are available from: https://microdata.worldbank.org/index.php/catalog/2734/related-materials. The NASQ module was fielded along with the core survey components in the Wave 3 or the Post-Harvest visit survey. Participants included informants, typically biological parents, of children ages 1−18 included in the General Household Survey (GHS) fielded by the National Bureau of Statistics (NBS) in 2015−2016. The GHS was reviewed and received local ethical approval, and participants provided informed consent. No other participant inclusion or exclusion criteria, beyond the general GHS procedures, were applied to data used in this analysis. The field workers collecting data for the GHS Wave 3 data were trained at the NBS office in Abuja, Nigeria, with the aid of audio−visual medium on each item of the questionnaires. They were also observed to conduct at least one independent interview with the questionnaires before being deployed to the field for data collection. These analyses used publicly available data with the permission of NBS and did not require an institutional review board (IRB) review. NASQ items are included in Supplement 1 and additional development details are included in Supplement 2.

### Measures

The NASQ tool was developed through a systematic selection of items common across existing autism screening tools (Aebi et al., 2012; Eaves et al., 2006; Juneja et al., 2014; Kakooza-Mwesige et al., 2014; Lord et al., 1994; Mung’ala-Odera et al., 2004; Silberberg et al., 2013; Skuse et al., 2004; Vats et al., 2018). The items were reviewed by Drs. Bakare and Omigbodun who are ASD clinical experts working in Nigeria. All initially identified items were rated as “very important,” “somewhat important,” and “not important.” Items were revised and deleted/modified based on the feedback from the Nigeria team. A field team in Kilif, Kenya, comprised of clinicians who have administered parent-report measures of ASD, were asked to draw on their field experience and provide a qualitative review of each item, including feasibility of administering items for low literacy population. The final selection of items was carried out to ensure coverage of the *DSM-5* criteria and associated symptom exemplars.

The final NASQ questionnaire (Supplement 1) included 26 questions eliciting dichotomous (yes/no) responses. The first two questions assess the presence of expected levels of speech. The remaining 24 questions assess core autism symptoms, including eight questions that assess early social communication/interaction skills and 16 items evaluating restricted/repetitive behaviors. For this study, core autism item endorsements were summed to generate a total raw score with a range 0−24. Four questions that assess developmental concerns were also collected as part of the larger national survey. These questions were used to describe the sample. In English, the questions were: (1) Are you worried about [Name’s] language and communication development? (2) Are you worried about [Name’s] relationship with peers? (3) Are you worried about [Name’s] development and use of hands and limbs?, and (4) Are you worried about [Name’s] odd or repetitive behavior? A positive response to any of the four questions was coded as a developmental concern (any developmental concern).

### Procedure

Data for this study, including the NASQ and developmental concern questions, were collected along with other information included in the third wave of the GHS panel. The GHS panel is the result of a partnership that the NBS has established with the Federal Ministry of Agriculture and Rural Development (FMA&RD), the National Food Reserve Agency (NFRA), the Bill and Melinda Gates Foundation (BMGF), and the World Bank (WB). The baseline GHS-Panel sample of 5000 households was designed to be representative at the national level as well as at the zonal level (both rural and urban).

### Statistical analyses

Descriptive statistics for demographic factors (age, sex, country zone, rural versus urban sector, and relationship to informant) and developmental concerns (any concern, language/communication, relationships with peers, motor development, odd or repetitive behavior) were computed to characterize the sample. Demographic factors and screening questionnaire total scores were compared between children with and without any developmental concern using independent samples *t*-tests and chi-square tests, where appropriate. Exploratory factor analyses were conducted with randomly split exploratory (*n* = 6207) and confirmatory (*n* = 6104) sub-samples. After identifying plausible 2−7 exploratory factor solutions, exploratory structural equation models (ESEM) using WLSMV estimation and specifying from one to seven factors (Asparouhov & Muthén, 2009; Marsh et al., 2014) were estimated using geomin rotation in both sub-samples. Once the best-fitting ESEM models were identified in the exploratory sample, equivalent CFA models were estimated with cross-loadings identified from ESEM models specified in the CFA framework.

The most parsimonious model derived from factor analysis was used to examine measurement invariance (Chiorri et al., 2016; Marsh et al., 2014; Tomas et al., 2014) across age groups (ages 1−6, 7−12, and 13−18) and sex (male, female). These analyses examine whether NASQ factors are measured consistently across age and sex. Consistent measurement across age is particularly important to ensure that the measure can be used to track symptoms over time. Reliability analyses including the classical test theory reliability (internal consistency and correct item-total correlations) (Streiner & Norman, 1995) and item response theory (IRT) analyses were conducted (Embretson & Reise, 2000; Hambleton et al., 1991; Reise et al., 1993). IRT analyses were conducted to evaluate whether NASQ total and subscale scores were able to differentiate individual differences in autism symptoms at different symptom levels—from very low to very high scores. Reliability at different score levels is a critical pre-condition for sensitivity to change because, for a measure to reliability capture change, it must reliably measure symptom levels at the score ranges where change is most likely to occur. This is often from very high scores (representing substantial symptom severity) to average scores (representing normative symptom levels). Additional statistical details can be found in Supplement 3.

Data preparation, descriptive analyses, internal consistency reliability, corrected item-total correlations, and bivariate correlations used SPSS, version 27 (IBM Corp, 2020). Factor, measurement invariance, and IRT were computed in MPlus version 8.5. Given the large sample size, analyses were over-powered for even small (*d* >.20) effect sizes. Therefore, where relevant, statistical significance was set at α=.05, two-tailed, and effect size magnitude or the size of differences in parameter estimates were emphasized.

### Community involvement

The lead author and co-authors work for a leading international organization advocating for the rights of people with ASD and their families. None of the authors are autistic, but the analysis was benefited through review from autistic advisory board members.

## Results

### Participant characteristics

**Figure 1:**
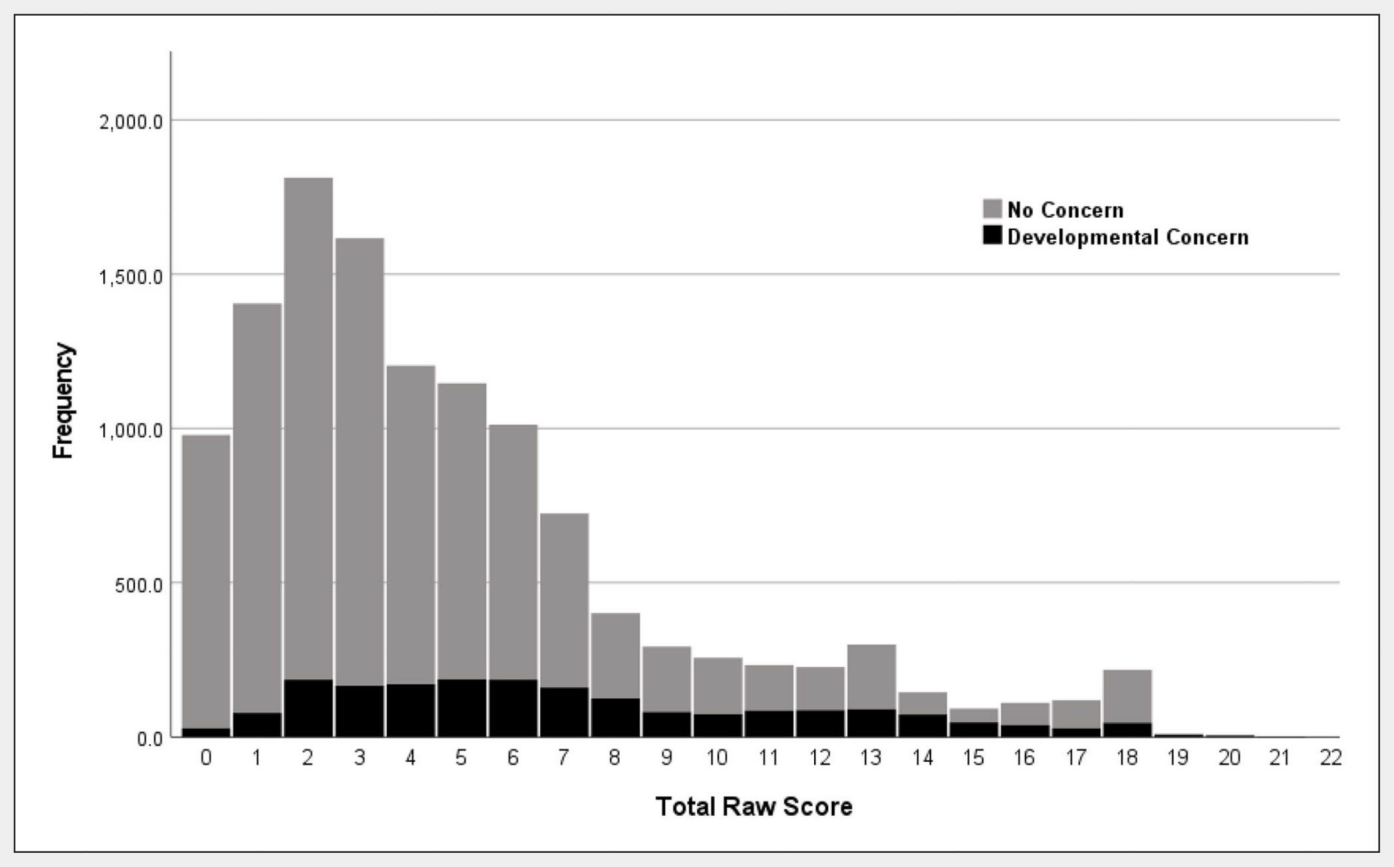
Nigerian Autism Screening Questionnaire total raw score distribution in individuals with and without developmental concern.

The final sample included 12,311 participants (ages 1−18), including 1272 participants ages 1−3 and 2160 participants ages 4−6. Most participants were biological children of the informant (86.7%), sex was shifted slightly toward males (53.4%), and a substantial minority had a developmental concern (15.9%) (Table 1). Geographic distributions were consistent with greater population in northern zones, with most (76.1%) living in northern zones and nearly three-quarters (73.2%) living in rural regions. NASQ total raw scores had a positively skewed distribution with the overall modal, median, and mean scores being 2, 4, and 4.9 (No Concern—Median = 3.0, M = 4.5, *SD* = 4.0; Developmental Concern—Median = 6.0, M = 7.4, *SD* = 4.6; Figure 1). Supplement 4 provides participant accounting and additional sample characteristics.

### Factor structure

Eigenvalues from exploratory factor analysis suggested a dominant first factor (11.8) with second through fifth factors being above 1.0 (4.6, 2.4, 1.3, and 1.1), respectively. While ESEM models suggested continued small improvements in fit through the seven-factor solution, the increases in confirmatory fit index (CFI) and Tucke−Lewis Index (TLI) and decreases in root mean square error of approximation (RMSEA) beyond three factors tended to be modest (≤.|01|) and subsequent factors were represented by small numbers of items (Supplement 5). The three-factor solution was easily interpreted based on the existing ASD factor literature, with a social communication/interaction factor strongly represented by items 5−10 (ex. gestures, eye contract, etc.), a repetitive sensory motor factor represented by verbal and motor stereotypies items 3−4 and 15−19 (ex. repetitive speech, spinning, etc.), and an insistence on sameness factor that included high loadings from items 11−14 and 20−26 related to obsessions and pre-occupations with facts and activities as well as sensory sensitivities and interest (Supplement 6). The latter factor mixes several sub-domains but appears generally consistent with *DSM*-5 criteria B2−B4 (American Psychiatric Association, 2013). CFA models including cross-loadings identified from the three-factor ESEM model in the exploratory sub-sample showed good fit in both the exploratory and confirmatory sub-samples. Based on the the dominant first factor, a single-factor solution was also considered for IRT analyses, particularly given that scoring for screening applications would most likely involve a single total raw score.

### Measurement invariance

The three-factor model described above showed strong evidence of measurement invariance of factor loadings and thresholds (scalar invariance) across sexes and age groups. In most cases, model fit improved slightly when restrictions were added to parameters (Supplement 7). Holding the means and variances equal also resulted in improved fit, suggested that the NASQ measures equivalently across males and females and from ages 1−18, even when measurement differences are estimated for the youngest children (age <3, *n* = 607; Supplement 7 bottom). Supplement 8 shows very little difference in factor loadings, means, and variances across age and sex, further confirming measurement equivalence. The only exception to this pattern was reductions with age in mean repetitive sensory motor behavior.

### Reliability

**Figure 2:**
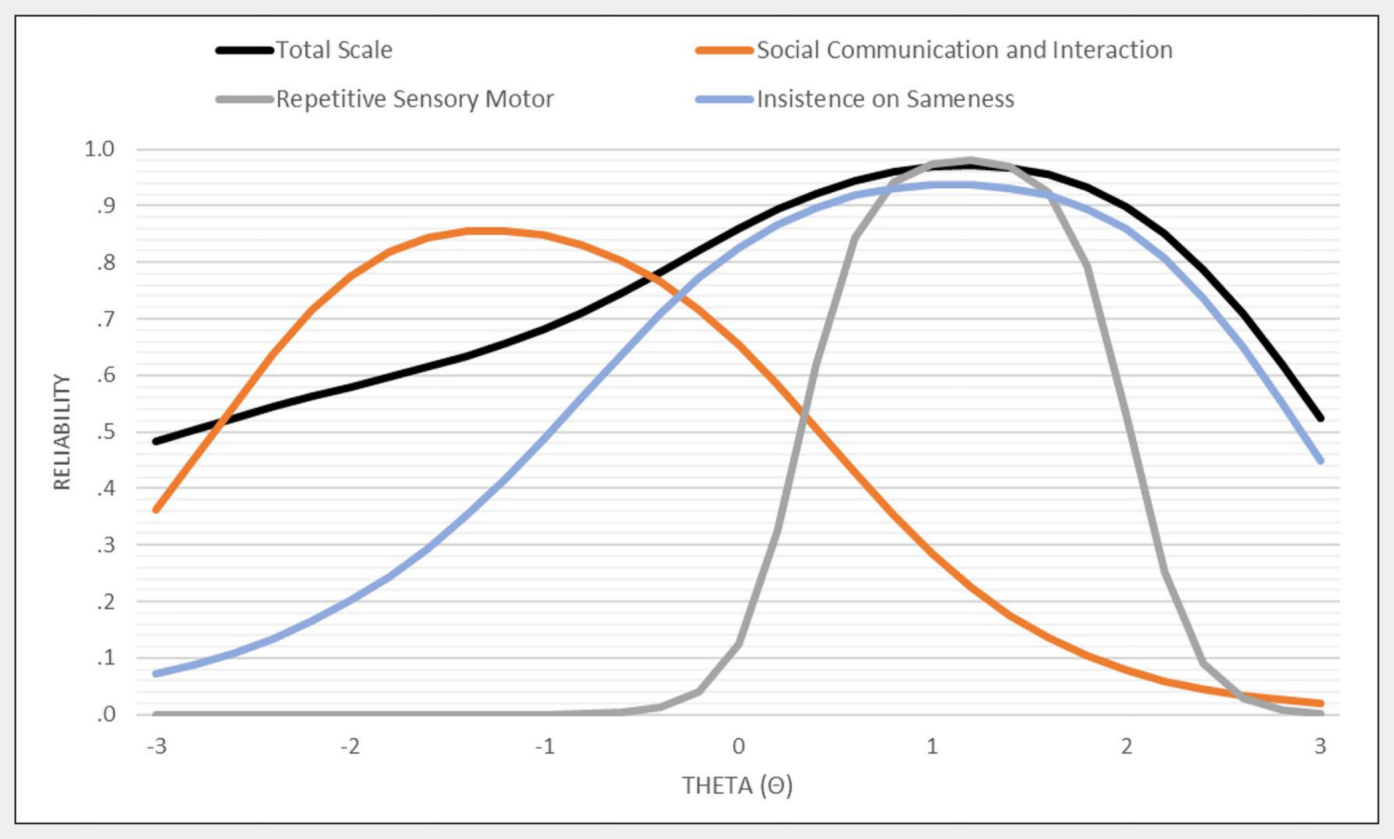
Conditional reliability for social communication interaction, repetitive sensory motor, and insistence on sameness factors and the total scale.

Classical test theory internal consistency reliability was very good in the full sample (α =.88) and across all age groups (α =.88−.89). Conditional reliability estimates indicated high reliability (≥.85) in the range from average scores (theta = 0) to very high scores (theta = 2.2) (Figure 2). IRT-derived theta scores showed strong correlations with NASQ total scores (*r* =.94). There was no evidence of differential measurement across sexes or age groups for total item information curves or for item characteristic curves (Supplements 9-12). However, items 5, 10, 12, 21, and 22 inquire about skills and problems that may not be applicable to very young children (age <3) and require evaluation in a larger sample of young children.

## Discussion

This study describes the initial psychometric evaluation of a newly developed, culturally sensitive autism symptom measure, the NASQ, as part of a nationally representative household survey in Nigeria, the most populous sub-Saharan African country. The present investigation takes an important first step by identifying that the NASQ is a reliable tool with good structural validity and might be useful as a screening and symptom tracking instrument in this resource-limited environment. Specifically, the results provide initial evidence of good psychometric characteristics of the NASQ in this large population sample. Specifically, a three-factor structure replicated across subsamples and was measurement invariant across sex and ages 1 to 18. The structure included separate social communication, repetitive sensory motor, and insistence on sameness dimensions, consistent with prior research indicating a major division between social and non-social indicators (Frazier et al., 2014; Gotham et al., 2008; Posserud et al., 2008) and secondary distinctions within non-social (repetitive behavior) indicators between repetitive sensory motor behaviors and restricted interests/insistence on sameness symptoms (Bishop et al., 2013; Evans et al., 2017; Frazier & Hardan, 2017). The three factors were positively correlated (*r* =.25−.62), all symptom endorsements were positively correlated, and the first component derived from exploratory factor analysis was large relative to subsequent components. This pattern suggests that a total raw score is a reasonable scoring approach, at minimum reflecting a useful formative latent variable (Lundqvist & Lindner, 2017).

Reliability analyses indicated that the total scale (all core autism symptom items) and identified sub-dimension scales had very good reliability for key regions of the latent trait (average to high scores). This indicates that NASQ total and subscale scores are likely to be quite precise in separating individual differences and it appears to do so equally well across males and females and throughout the 1−18 age range. The NASQ demonstrates comparable reliability to existing tool such has the SRS (Nguyen et al., 2019), AQ (Hoekstra et al., 2008), and SCQ (Berument et al., 1999). Together, clear factor structure, high reliability across a wide range of scores, and measurement invariance across sex and ages 1−18 are all crucial features that support potential use in future longitudinal studies, including clinical trials, and tracking of response to interventions. Additional validation work, potentially within the Nigerian GHS, is needed to evaluate possible screening efficiency and concurrent validity with other measures of autism symptoms and neurodevelopment.

## Limitations and future directions

The primary limitation of this study is the lack of gold-standard confirmation of ASD and the absence of other measures to evaluate convergent and discriminant validity. Additional limitations include the use of a dichotomous scale for the NASQ and the wide age spans in which the scale was implemented. Future work is needed to determine screening and diagnostic efficiency relative to gold-standard ASD diagnostic methods and to evaluate inter-rater reliability, temporal stability, and sensitivity to change of NASQ scores. Since the World Bank−supported GHS holds every 5 years, the next survey should incorporate a gold standard ASD diagnostic instrument and clinical confirmation to derive diagnostic accuracy (sensitivity and specificity) and determine the utility of the NASQ in screening and diagnostic contexts. Inspection of the NASQ score distribution in the present sample indicates that total raw scores between 8 and 11 are likely to be the most useful to explore as cut scores to inform screening and diagnostic judgments.

Future studies of the NASQ should more fully capture developmental concerns to examine discriminant validity. This work should also examine the potential for implementing an ordinal responses scale to enhance reliability and sensitivity to change, particularly since longitudinal studies and clinical trials need measures with good ability to measure across the score range to identify improvement or worsening in symptoms. While additional work is needed, including studies directly testing test−retest reliability and sensitivity to change, the present data suggest that the NASQ may have potential to track symptom levels in autistic individuals. Future near-term investigations should focus on a narrower age span to ensure that screening and diagnostic validity are maximized in younger ages where the scale will eventually be most useful. It is likely that some items, such as those inquiring about conversation, peer and imaginative play, and restricted interest will be less applicable in younger ages and may be removed from administration in very young children (age <3). Future directions should also explore the convergent and discriminant validity of the NASQ and develop clinically significant change norms to inform its use in intervention settings. If future research confirms the screening, diagnostic, and treatment tracking utility of the NASQ, it should be incorporated into the autism public health plan for Nigeria (Bakare et al., 2019), including implementation in primary pediatric care, schools, tertiary care, and intervention programs.

## Supplementary Material

Supplementary Material

## Figures and Tables

**Table I:** Demographic characteristics in the total sample and developmental concern sub-samples.

	Total	Ages 1−3:11	Ages 4−6:11	No concern	Developmental concern	*χ*^2^/*t* (p)	Cohen’s *d*
	M (*SD*)	M (*SD*)	M (*SD*)	M (*SD*)	M (*SD*)		
*N*	12,311	1272	2160	10,357	1954		
Age (range)	9.8 (4.6, 1−18)	2.5 (0.5)	5.1 (0.8)	9.8 (4.6, 1 −18)	9.7 (4.6, 1 −18)	1.1 (.270)	.03
Sex (n, % male)	6569 (53.4%)	1272 (53.7%)	1101 (51.0%)	5524 (53.3%)	1045 (53.5%)	0.1 (.907)	.01
Any developmental concern	1954 (15.9%)	232 (18.2%)	334 (15.5%)	−	−		
Worry about language/communication	1441 (11.7%)	169 (13.3%)	238 (11.0%)	−	−		
Worry about relationship with peers	1441 (11.7%)	158 (12.4%)	241 (11.2%)	−	−		
Worry about motor development	1183 (9.6%)	132 (10.4%)	194 (9.0%)	−	−		
Worry about odd or repetitive behavior	1026 (8.3%)	118 (9.3%)	168 (7.8%)	−	−		
Country zone						903.4 (<.001)	.56
Zone 1: North Central	2227 (18.1%)	193 (15.2%)	391 (18.1%)	1880 (18.2%)	347 (17.8%)		
Zone 2: North East	2507 (20.4%)	267 (21.0%)	435 (20.1%)	1667 (16.1%)	840 (43.0%)		
Zone 3: North West	3394 (27.6%)	426 (33.5%)	652 (30.2%)	3111 (30.0%)	283 (14.5%)		
Zone 4: South East	1321 (10.7%)	118 (9.3%)	219 (10.1 %)	1272 (12.3%)	49 (2.5%)		
Zone 5: South Central	1584 (12.9%)	141 (11.l%)	267 (12.4%)	1292 (12.5%)	292 (14.9%)		
Zone 6: South West	1278 (10.4%)	127 (10.0%)	196 (9.1%)	1135 (11.0%)	143 (7.3%)		
Rural sector	9008 (73.2%)	961 (75.6%)	1580 (73.1%)	7560 (73.0%)	1448 (74.1%)	1.0 (.310)	.02
Relationship to informant						14.6 (.012)	.07
Biological child	10,786 (87.6%)	1142 (89.8%)	1926 (89.1 %)	9108 (87.9%)	1678 (85.9%)		
Step child	108 (0.9%)	9 (0.7%)	15 (0.7%)	82 (0.8%)	26 (1.3%)		
Grandchild	791 (6.4%)	102 (8.0%)	166 (7.7%)	658 (6.4%)	133 (6.8%)		
Sibling	166 (1.3%)	5 (0.4%)	17 (0.8%)	127 (1.2%)	39 (2.0%)		
Niece/nephew	196 (1.6%)	8 (0.6%)	18 (0.8%)	161 (1.6%)	35 (1.8%)		
Other	264 (2.2%)	6 (0.5%)	15 (0.7%)	221 (2.1%)	43 (2.2%)		
NASQ total score (M, *SD*, range)	4.9 (4.2, 0−20)	5.2 (4.3, 0−19)	5.0 (4.2, 0−20)	4.4 (4.0)	7.3 (4.6)	−28.4 (<.001)	−.70

Note. *SD:* standard deviation; ASD = autism spectrum disorder. NASQ = Nigerian Autism Screening Questionnaire scored 0 = no problem, 1 = problem for 24 items evaluating ASD characteristics. For non-verbal children, items requiring the presence of speech were rated as 0.

## Lay abstract

Early intervention for individuals with autism spectrum disorder (ASD) is dependent on reliable methods for early detection. Screening for ASD symptoms is an important strategy in low- and middle-income countries that often lack adequate service infrastructure. This study aims to conduct preliminary evaluation of the psychometric properties of a tool developed and deployed in Nigeria called the Nigerian Autism Screening Questionnaire (NASQ). Results demonstrated that NASQ, when used as a community-based survey, has a clear factor structure with consistent measurement across age and sex, and that scores from below average to well above average are measured reliably. Future research is needed to examine the performance of this tool against confirmatory ASD diagnosis in screening and diagnostic contexts to further understand the utility and applicability of this tool in the resource-limited Nigerian setting.
