# Supplementary material for: Brief report: Validity and reliability of the Nigerian Autism Screening Questionnaire
Source: Autism. Author manuscript; Available in PMC 2022 Sep 6. (PMC7613535; doi:10.1177/13623613221080250)
Supplement: Supplementary Material [file EMS153571-supplement-Supplementary_Material.docx]

**Supplement 1. Nigeria Autism Screening Questionnaire.**

Note. Items are order by DSM-5 criteria. Codes after each question represent the order in which the question was asked on the national survey and correspond to V1-V26. Only items V3-V26 represent core autism symptoms as items V1 and V2 are related to the presence of expressive speech.

ITEM 1. Does [NAME] speak at all (can he or she make himself or herself understood in words; can he or she say any recognizable words)? (s4bq04)

Yes 1

No 2

ITEM 2. Does [NAME] speak normally for his/her age? (s4bq05)

Yes 1

No 2

**A1: Deficits in social-emotional reciprocity**

ITEM 5. Does [NAME] initiate a conversation with you? (s4bq08)

Yes 1

No 2

ITEM 11. Does [NAME] appear to be in his/her own world, no matter what he/she is doing (even when with other children)? (s4bq14)

Yes 1

No 2

**A2: Deficits in nonverbal communicative behaviors**

ITEM6. Can he/she communicate with you by using gestures? E.g. pointing with the index finger, nodding/shaking head for yes/no etc. (s4bq09)

Yes 1

No 2

ITEM 7. Does [NAME] smile back when you smile at them? (s4bq10)

Yes 1

No 2

ITEM 8. Does [NAME] maintain eye contact when talking to people? (s4bq11)

Yes 1

No 2

ITEM 9. Does [NAME] show the typical range of facial expressions? For instance, does he/she smile when happy? Show sadness when unhappy? Express surprise when something unexpected happens? (s4bq12)

Yes 1

No 2

**A3: Deficits in developing, maintaining, and understanding relationships**

ITEM 10. Does [NAME] participate in imaginative role playing like (cooking play)/dolls/clay/telephone/toy gun/motor car OR ‘teacher-student’, ‘thief-police’, 'mother-child', etc. with other kids interactively? (s4bq13)

Yes 1

No 2

ITEM 12. Does [NAME] prefer to play alone rather than joining his peers? (s4bq15)

Yes 1

No 2

**B1: Stereotyped or repetitive motor movements**

ITEM 3. Does [NAME] often repeat the same word or phrase over and over again in the same manner? (s4bq06)

Yes 1

No 2

ITEM 4. Does [NAME] repeat what you say? Copy your speech or the speech of others? (s4bq07)

Yes 1

No 2

ITEM 13. Does [NAME] have interests that are not typical for children his or her age, like an interest in objects like fans, light switches, radios, etc.? (s4bq16)

Yes 1

No 2

ITEM 14. Does [NAME] have any repetitive behavior? For instance, arranging toys or household objects in a specific manner over and over again. (s4bq17)

Yes 1

No 2

Does [NAME] keep on repeating any of the following? (s4bq17a-e)

ITEM 15. a) Flapping hands (moving hands up and down)

Yes 1

No 2

ITEM 16. b) Hand wringing (as if squeezing clothes)

Yes 1

No 2

ITEM 17. c) Toe-walking (walking on tip-toe)

Yes 1

No 2

ITEM 18. d) Swinging or spinning his/her body

Yes 1

No 2

ITEM 19. e) Making unusual finger or hand movements near his/her face?

Yes 1

No 2

**B2: Insistence on sameness, inflexible adherence to routines**

ITEM 20. Does [NAME] insist on sameness and actively resist any change in his/ her routines? (s4bq19)

Yes 1

No 2

**B3: Highly restricted, fixated interests that are abnormal in intensity or focus**

ITEM 21. Has [NAME] memorized unusual facts like schedules, history facts, or other sorts of facts that preoccupy him or her daily? (s4bq20)

Yes 1

No 2

ITEM 22. Is [NAME] ‘too obsessed’ with certain activities or interests beyond what you would expect for a child of his/her age? (s4bq21)

Yes 1

No 2

ITEM 23. Does [NAME] have excessive interest in odd or unusual things/activities which other children do not have? E.g. collecting sweet wrappers, nylon bags, piece of rope, pulling thread and rubber band etc. (s4bq22)

Yes 1

No 2

ITEM 24. Does [NAME] prefer to play with a particular part of a toy/object rather than the whole toy/object? For example: when playing with a toy car, only want to play with the tires and not the rest of the car. (s4bq23)

Yes 1

No 2

**B4: Unusual Sensory Experience**

ITEM 25. Is [NAME] hypersensitive or under sensitive to certain sensory inputs i.e. is indifferent to pain? Overly upset by certain sounds or too sensitive to light? (s4bq24)

Yes 1

No 2

ITEM 26. Does [NAME] show an unusual interest in certain sensory aspects of the environment? E.g. excessive touching or smelling of objects? (s4bq25)

Yes 1

No 2

**Supplement 2.** Nigeria Autism Screening Questionnaire (NASQ) development.

The NASQ was developed through a systematic selection of items common across existing autism screening tools. An initial review was conducted to identify the existent autism screening and diagnostic tools. Some of the reviewed scales included: the Ten Questions Questionnaire (TQQ) (Mung'ala-Odera et al., 2004); Neurodevelopmental Disorders Screening Tool (NDST) (Silberberg, Arora, Bhutani, Durkin, & Gulati, 2013); the Ten Questions Questionnaire plus (Kakooza-Mwesige et al., 2014); INCLEN diagnostic tool for autism spectrum disorder (INDT-ASD) (Juneja et al., 2014; Vats, Juneja, & Mishra, 2018); Developmental and Well-Being Assessment (DAWBA) (Aebi et al., 2012); Social Communication Questionnaire (SCQ) (Eaves, Wingert, Ho, & Mickelson, 2006); Developmental, Dimensional and Diagnostic Interview (3DI) (Skuse et al., 2004); the Autism Diagnostic Interview-Revised (ADI-R) (Lord, Rutter, & Le Couteur, 1994) with items suggested by experts from Nigeria reviewed by Drs. Bakare and Omigbodun who are ASD clinical experts working in Nigeria. All initially identified items were rated as ‘very important’, ‘somewhat important’ and ‘not important’. Items were revised and deleted/modified based on the feedback from the Nigeria team. A field team in Kilif, Kenya, comprised of clinicians who have administered parent-report measures of ASD, were asked to draw on their field experience, and provide a qualitative review of each item. In particular, the Kenyan field team was asked to advise on which items can easily be administered to parents with low literacy. The final selection of items was carried out to ensure coverage of the DSM-5 criteria and associated symptom exemplars.

The final NASQ questionnaire (Supplement 1) included 26 questions eliciting dichotomous (yes/no) responses. The first two questions assess the presence of expected levels of speech. The remaining 24 questions assess core autism symptoms, including eight questions that assess early social communication/interaction skills and 16 items evaluating restricted/repetitive behaviors. Six of the eight social communication/interaction items are framed in terms of demonstrating a skill and, therefore, are reverse scored so that item endorsement for all items reflects higher autism symptom levels. Three of the items are only applicable to individuals who generate speech. For non-verbal children, items requiring speech were left missing to provide more a accurate estimation of structural models, but were coded as 0 (“no” - not endorsed) for screening utility analyses. For the present study, core autism item endorsements were summed to generate a total raw score with a range 0-24.

***Additional Survey Questions***

Four questions that assess developmental concerns were also collected as part of the larger national survey. These questions were used to describe the sample. In English, the questions were: 1) Are you worried about [Name’s] language and communication development? 2) Are you worried about [Name’s] relationship with peers? 3) Are you worried about [Name’s] development and use of hands and limbs? and 4) Are you worried about [Name’s] odd or repetitive behavior? A positive response to any of the four questions was coded as a developmental concern (Any Developmental Concern). This was considered a conservative criterion because a large proportion (15.9%) reported Any Developmental Concern, implying that many individuals without ASD - including some without any significant developmental disability are included in this group.

**Supplement 3. Additional statistical methods.**

***Exploratory Factor Analyses***

Initial exploratory factor analyses with mean and variance-adjusted weighted least squares (WLSMV) estimation for categorical (dichotomous) indicators were conducted in the exploratory sub-sample using the 24 items assessing core autism traits (items 1 and 2 assessing normal speech production were not included as these are not part of the DSM-5 criteria). These analyses indicated plausible solutions up to 7 factors; the 8-factor model included a factor with only one salient loading.

***Exploratory Structural Equation Models***

Exploratory structural equation models were chosen as the initial factor structure method because, in behavioral research, items are expected to also load onto non-target factors. Setting the loading onto these non-target factors at 0 (as is done in traditional confirmatory factor analyses - CFA) results in poor overall fit as well as in biased parameter estimates (Asparouhov & Muthén, 2009; Marsh, Morin, Parker, & Kaur, 2014). ESEM sets item loadings on non-target factors close to zero, but still allows a degree of loading, a more realistic scenario for behavioral questionnaire data. For the present study, the comparative fit index (CFI), Tucker–Lewis index (TLI), root mean square error of approximation (RMSEA), and the 95% confidence interval of RMSEA were used to examine model fit. Previous empirical literature has suggested that CFI values >.92 and >.95 indicate adequate and excellent overall fit, respectively (Marsh, Hau, & Wen, 2004). TLI values >.95 have been suggested as good fit (Hu & Bentler, 1999). RMSEA values <.10 indicated marginal fit, <.08 and >.05 were judged as adequate fit, and <.5 were taken as indicative of good fit (Browne & Cudeck, 1993; Kline, 1998). To compare models, ∆CFI and ΔRMSEA with an increasing number of factors were used as additional metrics for choosing the optimal number of factors (Bentler, 1988; Hu & Bentler, 1999). Improvements of CFI >.01 and reductions of RMSEA >.01 suggest improved fit of nested models (Cheung & Rensvold, 2002).

***Measurement Invariance***

To examine measurement invariance (equivalence), a series of multi-group confirmatory factor analyses were computed using the theta parameterization and WLSMV estimation for categorical indicators. Multi-group confirmatory factor analyses permit simultaneously estimating factor structure in two pre-specified groups (ex. males and females). In the simplest model (baseline or configural model), separate estimates of factor loadings and thresholds/intercepts are generated for each group. This model assumes that the factor structure is not equivalent across groups. After this model is estimated, a series of increasingly more restrictive models representing gradually more equivalent structures between groups are estimated in a stepwise fashion. If, at any step, a substantial decrease in model fit is observed, then the groups are determined to be non-equivalent on the parameters fixed at that step. For example, if, in the second step (metric invariance), model fit decreases after fixing factor loadings across groups, then the groups do not have equivalent relationships between indicators and factors.

As stated above, the baseline model for these analyses estimated separate factor loadings and thresholds/intercepts. This model evaluated configural invariance – whether factors are represented by the same sets of items across groups – and served as a comparison to more restrictive models. The next model restricted factor loadings across groups to examine weak or metric invariance – whether groups show a similar pattern of relationships between items and the latent factors they measure. The next model restricted both factor loadings and intercepts/thresholds across groups to examine strong or scalar invariance. This model tested whether the constructs were measured on the same scale across groups. Residual variances for all items were fixed equal within and across groups to facilitate model estimation. Scalar measurement invariance is preferred prior to making group mean comparisons to ensure that any observed differences are truly a function of group membership. After establishing scalar measurement invariance, equivalence of factor means and variances was examined across age groups and sexes.

Model comparisons for measurement invariance analyses were based on empirical work indicating that a drop in CFI or TLI >.01 or an increase in RMSEA >.01 imply measurement non-equivalence (Chen, 2007; Cheung & Rensvold, 2002; Vandenberg & Lance, 2000). Chi-square difference tests were also computed between increasing restrictive models (Satorra & Bentler, 2010), but this metric is known to be overly sensitive and may identify trivial differences in model fit. Thus, for the present study, if any of the comparative fit indices fell beyond the cutoff, the more restrictive model was considered to have reduced fit.

***Item Response Theory Analyses***

Item response theory analyses used maximum likelihood estimation with robust standard errors and a logit link with the single factor mean and variance fixed to 0 and 1 respectively. Reliability estimates falling in the ranges .70 to .79, .80 to .89, and >.90 were considered fair, good, and excellent (Nunnally & Bernstein, 1994). Average corrected item-total correlations >=.30 were considered adequate or better (Streiner & Norman, 1995). Differential item and test functioning were evaluated by examining differences in item characteristic curves and test information curves across age groups and sexes.

***General Analytic Methods***

Factor, measurement invariance, item response theory, and latent profile analyses were computed in MPlus version 8.5. Support vector machine models were computed using R package e1071 (type=c-classification, svm-kernel=radial basis, cost=1, 10-fold cross-validation). Random forests were computed using R package randomForest (mtry=10, ntree=600). Logistic regression was computed using the glm function. ROC analyses were computed using the R package pROC (Robin et al., 2011). All R packages/procedures were implemented in version 3.6.1 (R Core Team, 2017) using R Studio version 1.2.1335.

**Supplement 4.** Participant accounting and additional sample characteristics.

Of the 12,366 participants included in the database, 47 did not complete any NASQ items and 8 were age<1; these individuals were excluded as the measure was not expected to be useful in this age range. In the final sample, a small proportion (4.6%) of children were rated as not producing speech or speaking normally on the first two NASQ items and had missing data on the three items requiring speech. No other missing data was present. Participants with and without any developmental concern were comparable in age, sex, rural sector, and relationship to informant. Although children with any developmental concern were more likely to reside in the North East sector and somewhat less likely to reside in the South East or South West zones. Unsurprisingly, children with any developmental concern had substantially higher average NASQ total raw scores.

**Supplement 5.** Model fit from factor analyses in the exploratory and confirmatory sub-samples.

| **Exploratory (n=6207)** | | | | | | | | | |
| --- | --- | --- | --- | --- | --- | --- | --- | --- | --- |
| **Model** | **Type** | **Par** | **Χ^2^** | **DF** | **RMSEA** | **95% CI** | **CFI** | **TLI** | **SRMR** |
| 1-Factor | CFA | 47 | 19020 | 253 | .109 | .108-.111 | .848 | .834 | .170 |
| 2-Factor | ESEM | 71 | 5934 | 229 | .063 | .062-.065 | .954 | .944 | .073 |
| 3-Factor | ESEM | 93 | 3330 | 207 | .049 | .048-.051 | .975 | .966 | .051 |
| 3-Factor | CFA | 62 | 3415 | 238 | .046 | .045-.048 | .974 | .970 | .064 |
| 4-Factor | ESEM | 114 | 2171 | 186 | .041 | .040-.043 | .984 | .976 | .038 |
| 5-Factor | ESEM | 134 | 1318 | 166 | .033 | .032-.035 | .991 | .984 | .029 |
| 6-Factor | ESEM | 153 | 771 | 147 | .026 | .024-.028 | .995 | .991 | .022 |
| 7-Factor | ESEM | 171 | 458 | 129 | .020 | .018-.022 | .997 | .994 | .016 |
| **Confirmatory (n=6104)** | | | | | | | | | |
| **Model** | **Type** | **Par** | **Χ^2^** | **DF** | **RMSEA** | **95% CI** | **CFI** | **TLI** | **SRMR** |
| 1-Factor | CFA | 47 | 18916 | 253 | .110 | .109-.111 | .832 | .816 | .172 |
| 2-Factor | ESEM | 71 | 6595 | 229 | .067 | .066-.069 | .943 | .931 | .082 |
| 3-Factor | ESEM | 93 | 3882 | 207 | .054 | .052-.055 | .967 | .956 | .058 |
| 3-Factor | CFA | 62 | 3635 | 238 | .048 | .047-.050 | .969 | .964 | .066 |
| 4-Factor | ESEM | 114 | 2579 | 186 | .046 | .044-.047 | .978 | .968 | .043 |
| 5-Factor | ESEM | 134 | 1453 | 166 | .036 | .034-.037 | .988 | .981 | .032 |
| 6-Factor | ESEM | 153 | 963 | 147 | .030 | .028-.032 | .993 | .986 | .025 |
| 7-Factor | ESEM | 171 | 676 | 129 | .026 | .024-.028 | .995 | .989 | .021 |

Note. CFA=confirmatory factor analysis. CFA is equivalent to ESEM for 1-factor solutions. ESEM=Exploratory Structural Equation modeling. DF=degrees of freedom. CFI=Comparative Fit Index. TLI=Tucker-Lewis Index. RMSEA=Root Mean Square Error of Approximation. 95% CI=95 percent confidence interval of the RMSEA. SRMR=standardized root mean residual.

***Interpretation***

ESEM models showed small improvements in fit through the seven-factor solution. However, the increases in CFI and TLI and decreases in RMSEA beyond three factors tended to be modest (≤|.01|) and subsequent factors were represented by small numbers of items. Therefore, along with interpretability considerations, the above results support retention of a three-factor solution.

**Supplement 6.** Standardized factor loadings derived from the three-factor exploratory structural equation model in the total sample (N=12,311).

|  | **F1: Social Communication and Interaction** | **F2: Repetitive**  **Sensory Motor** | **F3: Insistence on Sameness** |
| --- | --- | --- | --- |
| V3: Repeating words | .41 | **.64** | -.15 |
| V4: Copy others speech | **.53** | **.60** | -.14 |
| V5: Conversation | **.74** | .09 | -.12 |
| V6: Use gestures | **.79** | .12 | -.04 |
| V7: Smile back | **.92** | -.11 | -.09 |
| V8: Eye contact | **.74** | -.11 | .01 |
| V9: Typical expressions | **.76** | -.49 | .30 |
| V10: Imaginative play | **.59** | -.17 | .27 |
| V11: In own world | .16 | .09 | **.59** |
| V12: Play alone | .06 | .07 | **.69** |
| V13: Atypical interests | .17 | .02 | **.71** |
| V14: Repetitive arranging | .14 | .30 | **.56** |
| V15: Hand flapping | -.07 | **.78** | .26 |
| V16: Hand wringing | -.06 | **.80** | .25 |
| V17: Toe walking | -.07 | **.83** | .19 |
| V18: Spinning | -.05 | **.88** | .15 |
| V19: Hand/finger movements | -.08 | **.81** | .23 |
| V20: Same routines | .03 | .25 | **.59** |
| V21: Pre-occupation with facts | .01 | .10 | **.71** |
| V22: Obsessed with activities | .02 | .08 | **.73** |
| V23: Odd interest | -.02 | .20 | **.70** |
| V24: Play with part of object | -.01 | .16 | **.75** |
| V25: Hypersensitive | .03 | .05 | **.82** |
| V26: Unusual sensory interest | .03 | -.01 | **.87** |

Note. V1=Speak at all and V2=Speak normally were highly correlated (.87) and assess language production, which is not a core feature of autism. Further, preliminary exploratory factor analyses indicated that these items had low communality with other items. Therefore, these items were excluded from the factor analysis. F=Factor. Factor loadings are based on maximum likelihood estimation with Geomin rotation. Factor loadings ≥.50 are bold. Factor labels are based on the pattern of major loadings and are provided for descriptive purposes. The factor correlations were: F1-F2 r=.28, F1-F3 r=.25, F2-F3 r=.62. Removing zone 2 (n=2,507) which had very high levels of developmental concern did not substantively alter the factor structure – largest |Δ| factor loading = .07.

***Interpretation***

Standardized loadings from the three-factor solution indicate interpretable social communication/ interaction, repetitive stereotypies, and insistence on sameness. The social communication/interaction factor was strongly represented by items 5-10 (ex. gestures, eye contract, etc.). The repetitive sensory motor factor was represented by verbal and motor stereotypies items 3-4 and 15-19 (ex. repetitive speech, spinning, etc.). The insistence on sameness factor included high loadings from items 11-14 and 20-26 related to obsessions and pre-occupations with facts and activities as well as sensory sensitivities and interest. The insistence on sameness factor is generally consistent with DSM-5 criteria B2-B4.

**Supplement 7. Measurement invariance model comparisons for sex and age groups.**

| **Sex (M, F)** | | | | | | | | | | | | |
| --- | --- | --- | --- | --- | --- | --- | --- | --- | --- | --- | --- | --- |
|  | *Fit* | | | | | | *Difference Testing* | | | | | |
| Model | Par | Χ^2^ | DF | RMSEA | CFI | TLI | Χ^2^ | DF | p | ΔRMSEA | ΔCFI | ΔTLI |
| Configural | 106 | 7816.3 | 494 | .049 | .969 | .965 |  |  |  |  |  |  |
| Metric | 83 | 6763.3 | 517 | .044 | .973 | .972 | 23.9 | 23 | .409 | +.005 | +.004 | +.007 |
| Scalar | 62 | 6828.8 | 538 | .044 | .973 | .972 | 30.0 | 21 | .091 | .000 | .000 | .000 |
| Means Equal | 59 | 6597.9 | 541 | .043 | .974 | .974 | 3.64 | 3 | .303 | +.001 | +.001 | +.002 |
| Means and Variance Equal | 53 | 5482.8 | 547 | .038 | .979 | .979 | 8.77 | 9 | .459 | +.006 | +.006 | +.007 |
| **Age (1-6, 7-12, 13-18)** | | | | | | | | | | | | |
|  | *Fit* | | | | | | *Difference Testing* | | | | | |
| Model | Par | Χ^2^ | DF | RMSEA | CFI | TLI | Χ^2^ | DF | p | ΔRMSEA | ΔCFI | ΔTLI |
| Configural | 159 | 8142.6 | 741 | .049 | .969 | .965 |  |  |  |  |  |  |
| Metric | 113 | 6948.9 | 787 | .044 | .974 | .973 | 82.9 | 46 | .001 | +.005 | +.005 | +.008 |
| Scalar | 71 | 7164.9 | 829 | .043 | .973 | .973 | 248.0 | 42 | <.0001 | +.001 | -.001 | .000 |
| Means Equal | 65 | 6990.4 | 835 | .042 | .974 | .974 | 34.8 | 6 | <.0001 | +.001 | +.001 | +.001 |
| Means and Variance Equal | 59 | 5786.1 | 841 | .038 | .979 | .979 | 23.0 | 12 | .028 | +.004 | +.005 | +.005 |
| **Age (1-2, 3-6, 7-12, 13-18)** | | | | | | | | | | | | |
|  | *Fit* | | | | | | *Difference Testing* | | | | | |
| Model | Par | Χ^2^ | DF | RMSEA | CFI | TLI | Χ^2^ | DF | p | ΔRMSEA | ΔCFI | ΔTLI |
| Configural | 212 | 7900.3 | 988 | .048 | .971 | .967 |  |  |  |  |  |  |
| Metric | 143 | 6835.5 | 1057 | .042 | .976 | .975 | 140.3 | 69 | <.0001 | -.006 | +.005 | +.008 |
| Scalar | 80 | 7068.0 | 1120 | .042 | .975 | .975 | 292.8 | 63 | <.0001 | .000 | -.001 | .000 |
| Means Equal | 71 | 6902.3 | 1129 | .041 | .976 | .976 | 37.7 | 9 | <.0001 | -.001 | +.001 | +.001 |
| Means and Variance Equal | 62 | 5803.1 | 1138 | .036 | .980 | .981 | 28.6 | 18 | .0534 | -.005 | +.004 | +.005 |

Notes: CFI, comparative fit index; TLI, Tucker-Lewis index; RMSEA, root mean square error of approximation.

***Interpretation***

Measurement invariance analyses indicate that the NASQ factors are measuring consistently across ages 1-18 (even when ages 1-2 are broken out) and across males and females.

**Supplement 8. Standardized factor loading, mean, and variance differences between males and females and age groups (1-6, 7-12, and 13-18).**

|  | **F1 Loadings** | **F2 Loadings** | **F3 Loadings** | **F1 Loadings** | **F2 Loadings** | **F3 Loadings** |
| --- | --- | --- | --- | --- | --- | --- |
|  | ΔMale-Female | ΔMale-Female | ΔMale-Female | Largest \|Δ\| | Largest \|Δ\| | Largest \|Δ\| |
| V3: Repeating words | -.006 | .007 |  | .055 | .052 |  |
| V4: Copy others speech | -.017 | -.010 |  | .039 | .067 |  |
| V5: Conversation | -.011 |  |  | .082 |  |  |
| V6: Use gestures | .021 |  |  | .036 |  |  |
| V7: Smile back | .021 |  |  | .018 |  |  |
| V8: Eye contact | .033 |  |  | .025 |  |  |
| V9: Typical expressions | .026 |  |  | .119 |  |  |
| V10: Imaginative play | .042 |  |  | .020 |  |  |
| V11: In own world |  |  | -.020 |  |  | .019 |
| V12: Play alone |  |  | -.019 |  |  | .061 |
| V13: Atypical interests |  |  | -.009 |  |  | .024 |
| V14: Repetitive arranging |  |  | -.010 |  |  | .050 |
| V15: Hand flapping |  | .004 |  |  | .015 |  |
| V16: Hand wringing |  | .006 |  |  | .015 |  |
| V17: Toe walking |  | .000 |  |  | .019 |  |
| V18: Spinning |  | .005 |  |  | .018 |  |
| V19: Hand/finger movements |  | -.006 |  |  | .011 |  |
| V20: Same routines |  |  | .036 |  |  | .023 |
| V21: Pre-occupation with facts |  |  | .013 |  |  | .019 |
| V22: Obsessed with activities |  |  | .001 |  |  | .022 |
| V23: Odd interest |  |  | -.003 |  |  | .015 |
| V24: Play with part of object |  |  | .003 |  |  | .019 |
| V25: Hypersensitive |  |  | .015 |  |  | .024 |
| V26: Unusual sensory interest |  |  | .008 |  |  | .019 |
|  | **F1 Means** | **F2 Means** | **F3 Means** | **F1 Means** | **F2 Means** | **F3 Means** |
|  | ΔMale-Female | ΔMale-Female | ΔMale-Female | Largest Δ  ages  1-6 vs. 13-18 | Largest Δ  ages  1-6 vs. 13-18 | Largest Δ  ages  1-6 vs. 13-18 |
|  | -.047 | -.062 | -.032 | .013 | -.761 | -.075 |
|  | **F1 Variances** | **F2 Variances** | **F3 Variances** | **F1 Variances** | **F2 Variances** | **F3 Variances** |
|  | ΔMale-Female | ΔMale-Female | ΔMale-Female | Largest \|Δ\| | Largest \|Δ\| | Largest \|Δ\| |
|  | +.108 | -.046 | -.004 | .080 | .650 | .031 |

***Interpretation***

Factor loading and variance differences across age and sex are minimal (very close to 0), supporting the observation that the NASQ is measuring highly similarly across age and sex groups. Mean differences are also minimal with the exception of decreases in F2: Repetitive Sensory Motor behavior with age.

**Supplement 9. Additional results regarding conditional reliability across NASQ scales.**

Early social communication/interaction items measured best at lower score ranges because these items are positively worded statements asking about the development of early life social skills. In contrast, repetitive sensory motor and insistence on sameness behaviors measured best in the upper score ranges, as these behaviors tend to be very infrequent in non-ASD cases. There was no evidence of differential measurement across sexes or age groups as the total information curves (conditional reliability) were very similar (Supplement 10) and the item characteristic curves tended to be nearly identical for nearly all items (Supplements 11 and 12). The only minor exception was slightly more reliable measurement of social communication/interaction skills in males.

**Supplement 10. Conditional reliability for the total scale, separately in males and females (panel a) and in age groups (panel b).**

***Interpretation***

Reliability was good across below average, average, above average, and high score levels and was consistent between males and females and across ages.

**Supplement 11. Male and female item characteristic curves for three representative items, one each from the social communication and interaction, repetitive sensory motor, and insistence on sameness factors.**


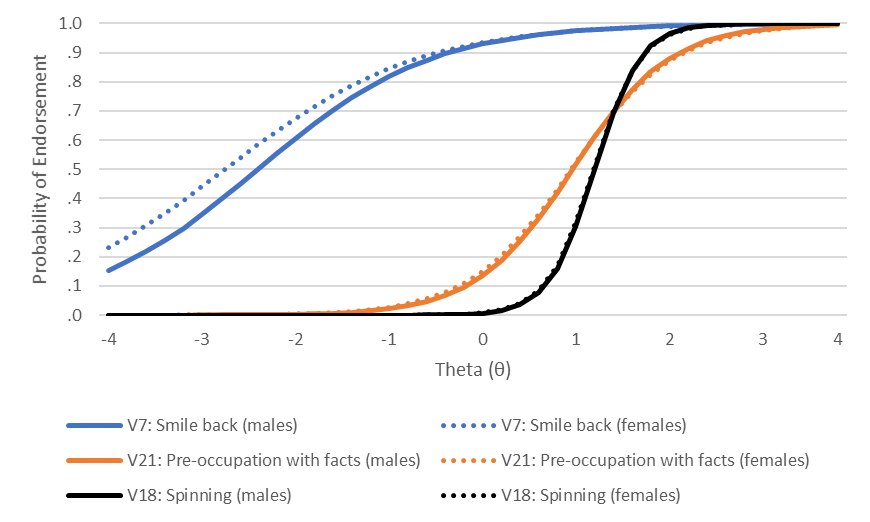


***Interpretation***

Item response patterns were highly consistent across all items between males and females.

**Supplement 12. Item characteristic curves across the three age groups for three representative items, one each from the social communication and interaction, repetitive sensory motor, and insistence on sameness factors.**

***Interpretation***

Item response patterns were highly consistent across all three age groups.

**References**

Asparouhov, T., & Muthén, B. (2009). Exploratory structural equation modeling. *Structural Equation Modeling, 19*, 397-438.

Bauer, D. J., & Curran, P. J. (2004). The integration of continuous and discrete latent variable models: Potential problems and promising opportunities. *Psychological Methods, 9*(1), 3-29.

Bentler, P. M. (1988). Comparative fit indexes in structural equation models. *Psychological Bulletin, 107*, 238-246.

Browne, M. W., & Cudeck, R. (1993). Alternative ways of assessing model fit. In K. A. Bollen & J. S. Long (Eds.), *Testing structural equation models*. Newbury Park, CA: Sage.

Chen, F. F. (2007). Sensitivity of goodness of fit indexes to lack of measurement invariance. *Structural equation modeling, 14*(3), 464-504.

Cheung, G. W., & Rensvold, R. B. (2002). Evaluating goodness-of-fit indexes for testing measurement invariance. *Structural Equation Modeling: A Multidisciplinary Journal, 9*(2), 233-255.

Hu, L., & Bentler, P. (1999). Cutoff criteria for fit indexes in covariance structure analysis: Conventional criteria versus new alternatives. *Structural equation modeling, 6*, 1-55.

Kline, R. B. (1998). *Principles and practice of structural equation modeling*. New York: Guilford.

Marsh, H. W., Hau, K. T., & Wen, Z. (2004). In search of golden rules: Comment on hypothesis-testing approaches to setting cutoff values for fit indices and dangers in overgeneralizing Hu and Bentler's (1999) findings. *Structural equation modeling, 11*, 320-341.

Marsh, H. W., Morin, A. J., Parker, P. D., & Kaur, G. (2014). Exploratory structural equation modeling: an integration of the best features of exploratory and confirmatory factor analysis. *Annu Rev Clin Psychol, 10*, 85-110. doi:10.1146/annurev-clinpsy-032813-153700

Morin, A. J. S., Maïano, C., Nagengast, B., Marsh, H. W., Morizot, J., & Janosz, M. (2011). Growth mixture modeling of adolescents trajectories of anxiety: The impact of untested invariance assumptions on substantive interpretations. *Structural Equation Modeling, 18*, 613-648.

Petras, H., & Masyn, K. (2010). General growth mixture analysis with antecedents and consequences

of change. . In D. W. A.R. Piquero (Ed.), *Handbook of Quantitative Criminology* (pp. 69-100). New York, NY: Springer.

Satorra, A., & Bentler, P. M. (2010). Ensuring Positiveness of the Scaled Difference Chi-square Test Statistic. *Psychometrika, 75*(2), 243-248. doi:10.1007/s11336-009-9135-y

Vandenberg, R. J., & Lance, C. E. (2000). A review and synthesis of the measurement equivalence literature: Suggestions, practices, and recommendations for organizational research. *Organizational Research Methods, 3*, 4-70.
